# Assessment of Antioxidant and Cytotoxicity Activities of Saponin and Crude Extracts of *Chlorophytum borivilianum*

**DOI:** 10.1155/2013/216894

**Published:** 2013-10-02

**Authors:** Mehdi Farshad Ashraf, Maheran Abd Aziz, Johnson Stanslas, Ismanizan Ismail, Mihdzar Abdul Kadir

**Affiliations:** ^1^Department of Agriculture Technology, Faculty of Agriculture, Universiti Putra Malaysia, 43400 Serdang, Selangor, Malaysia; ^2^School of Bioscience and Biotechnology, Faculty of Science and Technology, Universiti Kebangsaan Malaysia, 43600 Bangi, Selangor, Malaysia; ^3^Laboratory of Plantation Crops, Institute of Tropical Agriculture, Universiti Putra Malaysia, 43400 Serdang, Selangor, Malaysia; ^4^Department of Medicine, Faculty of Medicine and Health Sciences, Universiti Putra Malaysia, 43400 Serdang, Selangor, Malaysia

## Abstract

The present paper focused on antioxidant and cytotoxicity assessment of crude and total saponin fraction of *Chlorophytum borivilianum* as an important medicinal plant. In this study, three different antioxidant activities (2,2-diphenyl-1-picrylhydrazyl radical scavenging (DPPH), ferrous ion chelating (FIC), and **β**-carotene bleaching (BCB) activity) of crude extract and total saponin fraction of *C. borivilianum* tubers were performed. Crude extract was found to possess higher free radical scavenging activity (ascorbic acid equivalents 2578 ± 111 mg AA/100 g) and bleaching activity (IC_50_ = 0.7 mg mL^−1^), while total saponin fraction displayed higher ferrous ion chelating (EC_50_ = 1 mg mL^−1^). Cytotoxicity evaluation of crude extract and total saponin fraction against MCF-7, PC3, and HCT-116 cancer cell lines using 3-(4,5-dimethylthiazol-2-yl)-2,5 diphenyltetrazolium bromide (MTT) cell viability assay indicated a higher cytotoxicity activity of the crude extract than the total saponin fraction on all cell lines, being most effective and selective on MCF-7 human breast cancer cell line.

## 1. Introduction

Cancer is one of the most significant causes of human death. In a review, Hartwell [1] stated that 3000 plant species have been used for cancer treatment. Natural sources are the major part of anticancer agents [2], and the first study on anticancer agents of plant origin was carried out in the 1950s on vinca alkaloids, vinblastine, and vincristine [2]. Basically, plants are the major source of plant secondary metabolites. In addition to their food value, recent impact of plant secondary metabolites is on disease prevention in the form of antioxidant, antiviral, antibacterial, and anticancer compounds. Phytochemical compounds are secondary metabolites that are produced and used by plants for natural defense against environmental threats [3]. Antioxidant properties could be found in many phytochemical compounds, such as carotenoids and flavonoids [4]. Phytochemical screening should be simple and rapid with minimal equipment and selective techniques for screening certain compounds [5]. *Chlorophytum* species has an old history of medicinal use. In ancient Indian medicinal systems *C. borivilianum* is a remarkable herb for the treatment of rheumatism as well as having antidiabetic and spermatogenic properties [6]. Steroidal and triterpene saponins generally were shown to exhibit cytotoxicity activity on many cancer cell lines [7]. The steroidal and triterpene saponins found in *C. malayense* and ginseng as an example showed cytotoxicity against various cancer cells [8, 9]. A steroidal saponin of *C. borivilianum* showed cytotoxicity against HCT-116 and HT-29 human colon carcinoma cell lines [10].

The main objectives of this study are to evaluate antioxidant capacity of crude and total saponin extracts of *C. borivilianum* by spectrophotometric determination of free radical scavenging ability via 2,2-diphenyl-1-picrylhydrazyl radical scavenging (DPPH) radical scavenging assay, ferrous ion chelating activity (FIC), and lipid peroxidation inhibition effect by means of BCB assay. Also, cytotoxicity of total saponin and crude extracts was evaluated and screened against MCF-7 (breast), PC3 (prostate), and HCT-116 (colon) cancer cell lines. The reduction of viability of cells in different concentrations of both extracts was evaluated by using MTT assay. 

## 2. Materials and Methods

### 2.1. Plant Material

Fresh tubers of *C. borivilianum* were collected from Lanchang field in Pahang, Malaysia. The tubers were separated and washed with tap water containing detergent to remove soil and debris, cut into small pieces, washed with distilled water, and then dried in an oven at 45°C for 3 days until there was no change in weight. The dried samples were kept in a fridge at 4°C prior to the extraction and fractionation processes. Dried samples of in vitro tubers were prepared similarly. 

### 2.2. Extraction of Crude Extract from Tubers of *C. borivilianum*

Dried tubers were ground into a powdery form, and 20 g of the ground sample was macerated by soaking in 100 mL of a mixture containing 1 : 1 (v/v) ratio of methanol (MeOH) and dichloromethane (DCM). After 72 hours, the sample was filtered with Whatman filter paper no. 1. The residue from the extraction was soaked again with the same solvent mixture for 72 hours until the solvent layer became colorless. After filtration with Whatman no. 1 paper, the filtrates were mixed and the solvent was removed by using a rotary evaporator (Büch Rotavapor R-200) under reduced pressure in a water bath at a temperature not exceeding 55°C. The concentrated extract was transferred into a flask and left in an oven at 45°C to remove residual solvents. The final dried extract was weighed and stored at −20°C for future analysis.

### 2.3. Preparation of Total Saponin Fraction of *C. borivilianum* Tubers

The method of fraction was performed as described by Makkar et al. [3]. Ground dried tubers were defatted using distilled hexane for 24 hours by stirring with a magnetic stirrer. The solution was filtered through a Whatman filter paper no. 1, and in order to dry the hexane, defatted powders of tubers were placed overnight in an oven at 45°C. Ten grams of defatted samples was soaked in 100 mL of 50% aqueous methanol (MeOH) and mixed well overnight by using a magnetic stirrer at room temperature, and then the solution was centrifuged at 3000 g for 10 min and the supernatant collected. Extraction was repeated with the same solvent by overnight stirring on a magnetic stirrer, followed by centrifugation and collection of the supernatant. Both supernatants were combined and filtered through a Whatman filter paper no. 1. MeOH was removed from the solution using a rotary evaporator under vacuum at 40°C. Finally, concentrated total saponin in the aqueous phase was extracted by adding 100 mL equal volume of n-butanol (two times) through a separating funnel. In this step, the total saponin was moved from an aqueous phase to the butanol layer. For each time, butanol layer was kept. Both butanol layers were combined, and the solvent was evaporated under vacuum at a temperature not higher than 45°C using a rotary evaporator. In the final stage, the saponin was placed in a round bottomed flask and solvent was removed. Dried saponin fraction was dissolved in 5 mL of distilled water, freeze-dried, and kept at −20°C for further computation.

### 2.4. Methods for Screening Antioxidant Activity

#### 2.4.1. 2,2-Diphenyl-1-picrylhydrazyl (DPPH) Radical Scavenging Assay

DPPH radical scavenging assay was carried out according to the method of Leong and Shui [11]. Two milliliters of 0.15 mM DPPH (5.9 mg/100 mL methanol) was added to different dilutions of the crude extract and total saponin from tubers of mother plant. The reaction mixture was incubated for 30 min after which its absorbance was measured at 517 nm against methanol as both blank and negative control (1 mL MeOH in 2 mL of 0.15 mM DPPH). The experiment was conducted in triplicate. The decrease in absorbance was calculated as IC_50_ and expressed as mg ascorbic acid (AA) equivalents per 100 g of fresh material antioxidant capacity (AEAC) as follows [12]:

(1) $$\begin{array}{c} \text{AEAC }\left(\text{mg AA}/100\text{ g}\right)=\frac{{\text{IC}}_{50(\text{ascorbate})}}{{\text{IC}}_{50(\text{sample})}}\times 10^{5}. \end{array}$$

#### 2.4.2. Ferrous Ion Chelating (FIC) Assay

The ferrous ion chelating (FIC) ability assay was adapted from a method described by Gülçin [13]. Briefly, 1 mL of 0.1 mM (0.0278 g in 50 mL distilled water) FeSO_4_ was added to various dilutions of total saponin and crude extracts (1 mL), followed by the addition of 1 mL of 0.25 mM (0.0616 g in 25 mL distilled water) ferrozine. Both FeSO_4_ and ferrozine should be diluted 20 times before use. The reaction mixture was allowed to stand for 10 min at room temperature before the absorbance measurements were taken at 562 nm. Various dilutions of each extract were attempted in triplicate. The ferrous ion chelating property of extracts was calculated in percentage using the formula:

(2) $$\begin{array}{c} \text{Chelating activity }\left(\%\right)=\frac{A_{\text{control}}-A_{\text{sample}}}{A_{\text{control}}}\times 100, \end{array}$$

where *A*_control_ and *A*_sample_ are absorbance of the control and extract, respectively. The control contained 1 mL each of 75% methanol, FeSO_4_, and ferrozine. Also EC_50_ value of each extract can be obtained from the chelating activity (%) and compared. EC_50_ refers to the concentration of a drug which induces a response halfway between the baseline and maximum after some specified exposure time, which is also a measure of the drug potency. 

#### 2.4.3. *β*-Carotene Bleaching (BCB) Assay

A modified method of *β*-carotene bleaching (BCB) assay described by Kumazawa et al. [14] was employed in this study. The effectiveness of antioxidants in suppressing the action of radicals towards *β*-carotene was evaluated by monitoring the colour reduction by means of a spectrophotometer.

In order to create *β*-carotene/linoleic acid emulsion, 3 mL of *β*-carotene (5 mg/50 mL in chloroform) was added to linoleic acid (40 mg) and Tween 40 (400 mg). The chloroform in the mixture was removed with nitrogen gas, followed by the addition of 100 mL of oxygenated ultrapure water to prepare the *β*-carotene/linoleic acid emulsion. The emulsion was mixed well to get a homogeneous solution, and the initial absorbance of the emulsion at 470 nm was measured at time zero. Ultra-pure water was used as blank. 

Aliquots of the emulsion (3 mL) were added to the extracts in different concentrations (10, 50, and 100 *μ*L) and were then incubated at 50°C water bath for 60 min. The absorbance measurements were taken, and antioxidant activity was calculated according to the formula reported by Kumazawa et al. [14]. Four hundred milligrams of Tween 40 in 100 mL oxygenated water instead of extracts was used as control. In this assay, quercetin was used as a positive control. The degradation rate (DR) was measured using the following formula:

(3) $$\begin{array}{c} \text{Degradation rate }\left(\text{DR}\right)\text{ of }\beta \text{-carotene}=\ln\frac{A_{\text{initial}}/A_{\text{sample}}\;}{60}, \end{array}$$

where *A*_initial_ is the initial absorbance at time 0 and *A*_sample_ is the absorbance at 60 min.

The antioxidant activity (%AOA) was calculated using the following formula:

(4) Antioxidant  activity  (%AOA)  =[DRcontrol−DRsampleDRcontrol  ]×100,

where DR_control_ is the degradation rate of the control and DR_sample_ is the degradation rate of the sample.

### 2.5. Method for Cytotoxic Activity Screening

#### 2.5.1. Cell Culture and Cell Plating

Cytotoxicity assay was carried out in breast (MCF-7), prostate (PC3) and colon (HCT-116) cell lines. All cell lines were cultured in RPMI-1640 medium supplemented with 2 mM L-glutamine, 10% of heat-inactivated foetal bovine serum (FBS), 100 IU/mL of penicillin and 100 *μ*g mL^−1^ streptomycin in 25 cm^2^ tissue culture flasks at 37°C, and 5% CO_2_ in a humidified atmosphere. Once the cells had reached 70–80% confluency, they were washed with sterile phosphate buffer saline (PBS) and 1 mL of trypsin-EDTA was added into the flask and maintained for 5–10 minutes in the CO_2_ incubator at 37°C to allow the cells to detach from the flask. The cell was centrifuged, the supernatant was discarded, and the cell pellet was resuspended in culture medium. The cell density was determined using a haemocytometer. 

#### 2.5.2. MTT (3-[4,5-Dimethylthiazol-2-yl]-2, 5-Diphenyltetrazolium Bromide) Cell Viability Assay

The potential of extracts to induce growth inhibition on several cell lines was determined by using MTT cell viability assay as described by Jada et al. [15]. Cancer cells were seeded into each well of the 96-well microplate. The cells were incubated overnight at 37°C and 5% CO_2_ to allow the cells to attach to the wells before they were treated with various concentrations of crude and saponin extracts. 20 *μ*L of varying concentrations (100 *μ*g mL^−1^, 10 *μ*g mL^−1^, 1 *μ*g mL^−1^ and 0.1 *μ*g mL^−1^) of the extracts was obtained from the stock solutions (100 mg mL^−1^ in DMSO) in RPMI-1640 medium and added into each well to give four volumes of 200 *μ*L in each well of the microtitre plate. Concentration of extracts was tested in quadruplicate, and the control wells contained 200 *μ*L of medium only. The culture plates were incubated at 37°C and 5% (v/v) CO_2_ for 96 hours. After 96 hours of incubation, 50 *μ*L of MTT solution (2 mg mL^−1^ in PBS) was added into each well containing 200 *μ*L medium and incubated at 37°C for 4 hours. Excessive MTT was discarded and 100 *μ*L of DMSO was added to each well and gently shaken to dissolve the purple formazan crystals that were formed. Absorbance values of the formazan as a measure of viable cells was determined at 550 nm with a microplate reader. Semi-log dose-response curves (percentage growth versus concentrations) were constructed to obtain growth inhibition values (GI_50_, TGI, and LC_50_). 

## 3. Results and Discussion

### 3.1. DPPH Radical Scavenging Activity

Antioxidant activity of both crude and total saponin extracts was investigated using DPPH radical scavenging assay. This method evaluates the antioxidant activity based on the scavenging of stable DPPH radicals. 

DPPH is a compound composed of a nitrogen-free radical which is easily quenched by a free radical scavenger [12]. The scavenging reaction between DPPH and antioxidant compound (H-A) is due to the ability of antioxidants to change DPPH as a stable free radical to the DPPH-H (nonradical form). 

The formula can be written as

(5) (DPPH)+(H-A)→DPPH-H+(A)(Purple)(Yellow)

**Figure 1:**
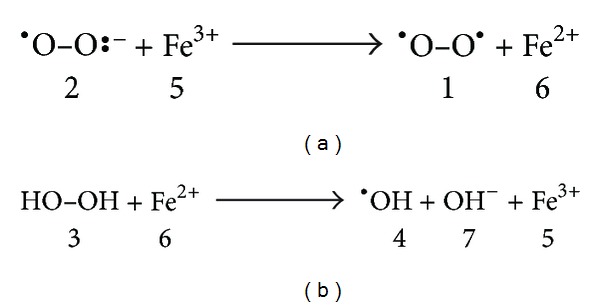
Haber-Weiss and Fenton reactions. (a) Fe^3+^ (5) is reduced to Fe^2+^ (6) by one-electron transfer from superoxide radical anion (2) to give dioxygen (1). (b) Fe^2+^ (6) is oxidized back to Fe^3+^ (5) by hydrogen peroxide (3) leading to the formation of hydroxyl radical (4) and hydroxide (7).

The rate of discoloration by extracts or antioxidant compounds indicates the potential of their scavenging in terms of hydrogen donating ability [16]. Commonly, reactive oxygen species (ROS) such as hydrogen peroxide (H_2_O_2_), hydroxyl radical (^∙^OH), and free radicals affect biological tissues due to oxidative stress [17]. Studies have proved that transition metals such as the cations of iron and copper have the ability to catalyze the formation of ROS like hydroxyl radicals (^∙^OH) [18] which is known as Haber-Weiss, and Fenton-type reactions [12] (Figure 1).

The lowest absorbance at 517 nm of reaction between DPPH and serial dilution of crude and total saponin extracts of mother plant tubers of *C. borivilianum *indicated higher free radical scavenging activity. Determination of antioxidant activity was presented in terms of AEAC and IC_50_ as the concentration of compound to obtain 50% scavenging of DPPH free radical. In this study, it was found that AEAC of crude extract was significantly higher than that of total saponin fraction (Table 1) indicating higher equivalent ascorbic acid or higher antioxidant activity. Studies on crude extract of *C. borivilianum* [19, 20] and *C. tuberosum* [21] discovered similar observation. Govindarajan et al. [19] reported, 100 *μ*g mL^−1^ concentration of ethanolic tuber extract of *C. borivilianum* was able to scavenge 84.51% DPPH. 

In this study, total saponin has higher IC_50_ than crude extract which indirectly indicate that crude extract has highly quenching capacity. Likewise, total saponin extract exhibited a lower AEAC (1062 ± 31 mg AA/100 g) than crude extract (2578 ± 11 mg AA/100 g) which means that more concentration of total saponin was required to scavenge 50% of the free radicals (440 ± 49 *μ*g mL^−1^) in contrast to crude extract (181 ± 34 *μ*g mL^−1^). 

### 3.2. Ferrous Ion Chelating (FIC) Activity

In this study, the inhibition of peroxidation of macromolecules by extracts of mother plant tubers of *C. borivilianum* was investigated by ferrous ions (Fe^2+^) chelating activity. It means that the extracts have an ability to compete with ferrozine in the process of chelating ferrous ions. 

A developed pathway for enhancing oxidative stability is essential since lipid and proteins were deteriorated by oxidative reactions [17]. Antioxidants are able to form chelates from the transition metal ions, which result in the repression of ^∙^OH generation, and inhibit deterioration process which is known as peroxidation of lipid, protein, and other biological molecules [12]. 

**Figure 2:**
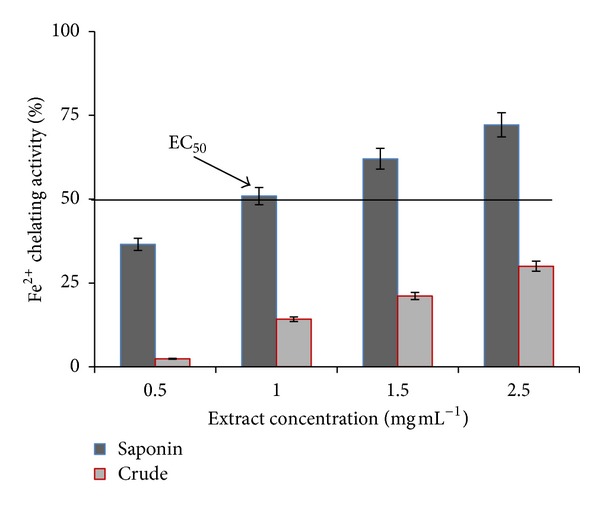
Ferrous ion chelating activity of total saponin and crude extracts of *C. borivilianum*. Data are expressed as means ± SD (*n* = 3).

In this study, the chelating abilities of the crude and total saponin extracts were 2.4% and 36.5% at 0.5 mg mL^−1^ and 30% and 72.2% at 2.5 mg mL^−1^, respectively (Figure 2). Analysis showed iron chelating property in both samples in a concentration-dependent manner. Total saponin in all concentrations (0.5, 1, 1.5, and 2.5 mg mL^−1^) showed high FIC values (*P* < 0.05) which indicate higher antioxidant activity. In this study, the EC_50_ value of total saponin was 1 mg mL^−1^, but crude extract showed >2.5 mg mL^−1^.

### 3.3. *β*-Carotene Bleaching (BCB) Activity

The antioxidant activity of crude and total saponin extracts of mother plant tuber of *C. borivilianum* was evaluated by the *β*-carotene bleaching (BCB) assay. Determination of antioxidant activity using the *β*-carotene linoleic acid bleaching method is based on the discoloration or bleaching of *β*-carotene caused by radicals released upon the oxidation of linoleic acid in the emulsion [22]. *β*-carotene bleaching ratio may decelerate in the presence of antioxidant [23] which is able to reduce the rate of chain reaction initiated during lipid peroxidation and transform the reactive end product to a more stable form. Lipid peroxidation activity in the *β*-carotene-emulsion systems was inhibited by antioxidants.

**Figure 3:**
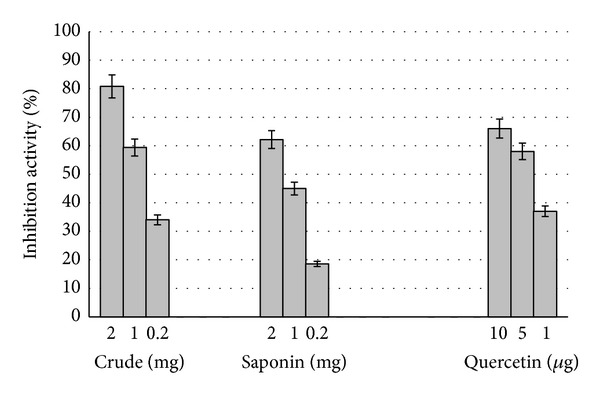
BCB antioxidant activity of crude and total saponin extracts from mother plant tuber of *C. borivilianum*.

**Figure 4:**
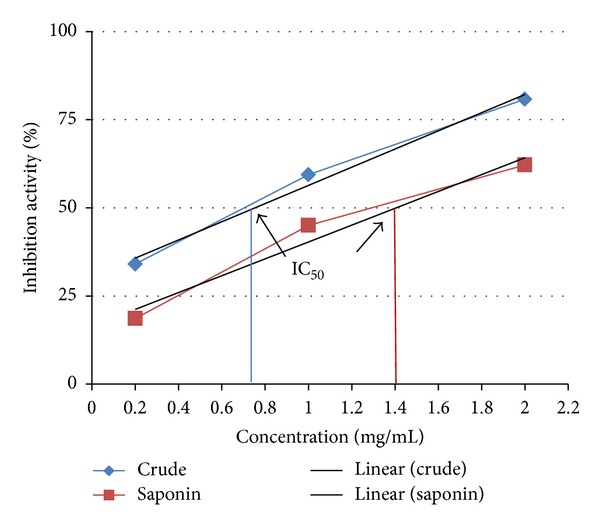
IC_50_ value in BCB assay of total saponin and crude extracts of mother plant tuber of *C. borivilianum*.

In this study, both extracts displayed potential of quenching linoleate free radicals which resulted from peroxidation of linoleic acid. The inhibition activity is certainly related to the mass of sample (Figure 3). The crude extract displayed stronger inhibition effect (80.6 ± 0.8%) at 2 mg compared to total saponin which showed 61 ± 1.14% at the same quantity. The lowest activity (18.6 ± 1.47%) was demonstrated by 0.2 mg total saponin in comparison to 34% with the same amount of crude extract. In terms of IC_50_, the crude and total saponin extracts showed 0.7 and 1.3 mg mL^−1^, respectively, which indicates higher antioxidant activity of crude extract (Figure 4).

One of the important biochemical processes is peroxidation of lipid that directly or indirectly led to diseases like diabetes, tumor, and so forth. Free radicals containing oxygen or reactive oxygen species (ROS) were formed from this biochemical process [24] and eliminated by antioxidant defense mechanisms.

Overall, the evaluation of free radical scavenging effect of various antioxidant foods and natural products has been determined by DPPH assay [25]. In the DPPH method, the scavenging effects of crude and total saponin extracts of *C. borivilianum* on the stable radical DPPH by converting it to the yellow colored DPPH-H increased with increasing concentrations of the extracts. However, IC_50_ of crude extract was less than that of total saponin or indicated 2.5-fold stronger antioxidant activity than that of total saponin (Table 1). Similar to DPPH method, antioxidant property in *β*-carotene emulsion was exhibited more in crude extract than total saponin (Figure 3). But total saponin showed higher chelating activity than crude extract (Figure 2). In this method, crude and total saponin interfered with the formation of ferrous and ferrozine complex suggesting that they have chelating activity and are able to capture ferrous (Fe^+2^) ion before ferrozine.

Several studies have shown antioxidant properties from different plant parts of *Chlorophytum* species [19–21, 24]. To our knowledge, there are no data regarding the antioxidant property of *C. borivilianum* total saponin. Saponin indeed occurs in many wild and cultivated plants, and triterpene saponin (total saponin) is supreme in cultivated crops compared to wild plants [26]. Since plants used in this study were cultivated types, therefore total saponin was determined and evaluated. 

In this study, total saponin was proven to have some antioxidant activity. Earlier, Gülçin et al. [27] observed that saponins derivatives isolated from *Hedera helix* L. (Araliaceae) showed profitable antioxidant activity and can be used as a source of natural antioxidant. Gülçin et al. [28] showed that saponin in *Leontice smirnowii* Trautv. (Berberidaceae) has strong and effective antioxidant activity based on DPPH radical scavenging and iron chelating activities. Screening showed that both crude and total saponin extracts displayed antioxidant activity which may relate to total phenolics concentration of extracts. Visavadiya et al. [29] reported a positive correlation between antioxidant activity and polyphenol and flavonoid contents of *C. borivilianum* root extract. 

### 3.4. Growth Inhibition Values (GI_**50**_, TGI, and LC_**50**_) of *C. borivilianum* Total Saponin Extracts on MCF-7, PC3, and HCT-116 Cell Lines

Growth inhibitory parameters (GI_50_, TGI, and LC_50_) of crude and total saponin extracts were measured by plotting semilog graphs (% growth versus concentration). The GI_50_ values of *C. borivilianum* crude extract on MCF-7, PC3, and HCT-116 cells were 7.3 ± 0.6 *μ*g mL^−1^, 23.7 ± 1.5 *μ*g mL^−1^, and 27 ± 1 *μ*g mL^−1^, respectively (Table 2). Meanwhile, the TGI values of the crude extract which represent the concentration required to completely halt the growth of treated cells were 24.7 ± 0.5 *μ*g mL^−1^, 51 ± 3.6 *μ*g mL^−1^, and 55.7 ± 0.6 *μ*g mL^−1^ for MCF-7, PC3, and HCT-116 cells, respectively. The range of LC_50_ values of crude extract which represent the concentration that kills 50% of treated cells was >100 *μ*g mL^−1^ in all treated cell lines.

Crude extract is considered active if the GI_50_ value <100 *μ*g mL^−1^ according to NCI guideline (http://www.cancer.gov/); crude extract of *C. borivilianum* showed strong activity and selectivity activity against MCF-7 tumor cell lines and moderate activity against PC3 and HCT-116. The activity was 3-4-fold selective towards MCF-7 cells. 

In Table 3, GI_50_ value of total saponin extract was 75 ± 0.5 *μ*g mL^−1^ on MCF-7 cell line whereas there was no activity found on PC3 and HCT-116. TGI and LC_50_ values in all cell lines were shown to be >100 *μ*g/mL. The results therefore indicate that total saponin extract had a selectively moderate cytotoxic effect on MCF-7 cell line while there was no cytotoxicity of total saponin against PC3 and HCT-116 cell lines.

However, in this study, total saponin did not show cytotoxicity activity except on MCF-7 cells. Meanwhile, crude extract compared to total saponin also showed stronger activity of more than 10-fold towards MCF-7 cells. Our results in the cytotoxicity assays are in agreement with previous finding of Acharya et al. [10, 30], which indicated that among the isolated steroidal saponins from the dried roots of *C. borivilianum, *the only active compound was borivilianoside H against colon tumor cell line. Triterpene (total saponin) and steroidal saponins were also able to stimulate apoptosis in tumor cells [31], which is also in accordance with our results. Further studies are needed to discern percentage of terpenoid saponins in the total saponin extracts and amount of borivilianoside H. 

Quercetin significantly inhibited human breast cancer cells (MCF-7 and MDA-MB231) [32]. Rahman et al. [33] mentioned the cytoprotective role of quercetin against oxidative stress which leads to protecting cells from free radical damage through antioxidant effect, motivating apoptotic cell death via prooxidant activity, and inhibiting tumourigenesis.

## Figures and Tables

**Table 1:** Free radical scavenging activity of crude and total saponin extract from mother plant tubers of *C. borivilianum*.

Sample	Antioxidant activity	
	IC_50_ (*μ*g mL^−1^)	AEAC (mg AA/100 g)^1,2^
Total saponin	440 ± 49^b^	1062 ± 31^b^
Crude extract	181 ± 34^a^	2578 ± 111^a^

Results are expressed as means ± SD (*n* = 3). For each column, values followed by different letter (a-b) are statistically significant (*P* < 0.05) as determined using ANOVA.

^1^IC_50 _of AA = 4.5 *μ*g mL^−1^.

^2^100 g fresh plant materials.

**Table 2:** Growth inhibition values (GI_50_, TGI, and LC_50_) of *C. borivilianum* crude extract on MCF-7, PC3, and HCT-116 cell lines.

Parameter (*μ*g mL^−1^)	Cell lines		
	MCF-7	PC3	HCT-116
GI_50_	7.30 ± 0.60	23.70 ± 1.50	27.0 ± 1.0
TGI	24.70 ± 0.50	51.0 ± 3.60	55.70 ± 0.60
LC_50_	>100	>100	>100

Values are expressed as mean ± SD obtained from 3 independent experiments.

**Table 3:** Growth inhibition values (GI_50_, TGI, and LC_50_) of *C. borivilianum* total saponin extract on MCF-7, PC3, and HCT-116 cell lines.

Parameter (*μ*g mL^−1^)	Cell lines		
	MCF-7	PC3	HCT-116
GI_50_	75 ± 0.5	>100	>100
TGI	>100	>100	>100
LC_50_	>100	>100	>100

Values are mean ± SD obtained from 3 independent experiments.
